# Mesenchymal stromal cells protect hepatocytes from lipotoxicity through alleviation of endoplasmic reticulum stress by restoring SERCA activity

**DOI:** 10.1111/jcmm.16338

**Published:** 2021-02-16

**Authors:** Linzhao Li, Xin Zeng, Zhenzhen Liu, Xuanming Chen, Lan Li, Ruixi Luo, Xiaohong Liu, Jie Zhang, Jingping Liu, Yanrong Lu, Jingqiu Cheng, Younan Chen

**Affiliations:** ^1^ Key Laboratory of Transplant Engineering and Immunology, NHFPC Regenerative Medicine Research Center West China Hospital, Sichuan University Chengdu China; ^2^ Medical College Guizhou University Guiyang China; ^3^ Center of Infectious Diseases West China Hospital of Sichuan University Chengdu China

**Keywords:** calcium homeostasis, hepatic steatosis, insulin resistance, mesenchymal stromal cells, SERCA

## Abstract

The aim of this study was to investigate how mesenchymal stromal cells (MSCs) modulate metabolic balance and attenuate hepatic lipotoxicity in the context of non‐alcoholic fatty liver disease (NAFLD). In vivo, male SD rats were fed with high‐fat diet (HFD) to develop NAFLD; then, they were treated twice by intravenous injections of rat bone marrow MSCs. In vitro, HepG2 cells were cocultured with MSCs by transwell and exposed to palmitic acid (PA) for 24 hours. The endoplasmic reticulum (ER) stressor thapsigargin and sarco/ER Ca^2+^‐ATPase (SERCA2)–specific siRNA were used to explore the regulation of ER stress by MSCs. We found that MSC administration improved hepatic steatosis, restored systemic hepatic lipid and glucose homeostasis, and inhibited hepatic ER stress in HFD‐fed rats. In hepatocytes, MSCs effectively alleviated the cellular lipotoxicity. Particularly, MSCs remarkably ameliorated the ER stress and intracellular calcium homeostasis induced by either PA or thapsigargin in HepG2 cells. Additionally, long‐term HFD or PA stimulation would activate pyroptosis in hepatocytes, which may contribute to the cell death and liver dysfunction during the process of NAFLD, and MSC treatment effectively ameliorates these deleterious effects. SERCA2 silencing obviously abolished the ability of MSCs against the PA‐induced lipotoxicity. Conclusively, our study demonstrated that MSCs were able to ameliorate liver lipotoxicity and metabolic disturbance in the context of NAFLD, in which the regulation of ER stress and the calcium homeostasis via SERCA has played a key role.

## 1 INTRODUCTION

Non‐alcoholic fatty liver disease (NAFLD) is characterized by abnormal lipid accumulation in hepatocytes in the absence of alcohol abuse, and can progress from simple steatosis to non‐alcoholic steatohepatitis (NASH), cirrhosis and ultimately cancer. In the last decade, the prevalence of NAFLD is strikingly increased, reaching 30% in adults in developed countries.^1^, ^2^ Although intense lifestyle change aiming at weight loss is the main therapy for hepatic steatosis, it is ineffective when NAFLD has progressed to NASH and further severe liver damage.

Mesenchymal stromal cells (MSCs) are multipotent stromal cells, showing promising therapeutic potentials in a spectrum of clinical settings, including heart failure, graft vs host disease (GvHD) and acute or chronic liver damage.^3^ Our previous studies have demonstrated that MSC transplantation ameliorated hyperlipidaemia in STZ‐induced diabetic rats.^4^ Nevertheless, how MSCs modulate metabolic balance and attenuate hepatic lipotoxicity in the context of NAFLD is still elusive.

The pathogenesis of NAFLD is hypothesized to begin with abnormal accumulation of lipids in the liver due to a stress condition such as saturated fatty acid (SFA) overexposure or insulin resistance, and several signalling mechanisms including the accumulation of reactive oxygen species, endoplasmic reticulum (ER) stress, and chronic inflammation are involved in this process.^1^, ^5^, ^6^

Endoplasmic reticulum performs important functions related to the synthesis, folding and transport of proteins and plays a critical role in lipid synthesis and Ca^2+^ homeostasis.^7^ Prolonged SFA overuptake leads to imbalance between ER protein load and ER folding, resulting in hyperactivation of unfolded protein response (UPR) and consequently activation of cell death pathways.^8^ Thus, any substance that opposes ER stress may have possible potential to treat NAFLD.

Endoplasmic reticulum calcium disequilibrium is considered to be one of the initial and pivotal events of ER stress‐mediated cell death.^9^ Herein, the disruption of cellular Ca^2+^ homeostasis is a hallmark of many ER‐related diseases and a key trigger of NAFLD/NASH as well. The sarco/endoplasmic reticulum (ER/SR) Ca^2+^ ATPase (SERCA) transports Ca^2+^ from the cytosol to the ER or SR lumen, maintaining the resting calcium concentration. Previous studies have demonstrated that SFAs inhibit the activity of SERCAs, which leads to calcium release from the ER lumen and a heavy calcium overload in the cytosol, ultimately resulting in the disruption of ER homeostasis and cell function.^10^, ^11^

Here, we aimed to evaluate the potential therapeutic effects of MSCs on hepatic lipotoxicity using high‐fat diet (HFD)–fed rats in vivo and palmitic acid (PA)–treated hepatocytes in vitro*,* and explore the underlying mechanisms focusing on the regulation of ER stress and SERCA activity by MSCs.

## 2 MATERIALS AND METHODS

### 2.1 In vivo

#### 2.1.1 Experimental animals

**FIGURE 1:**
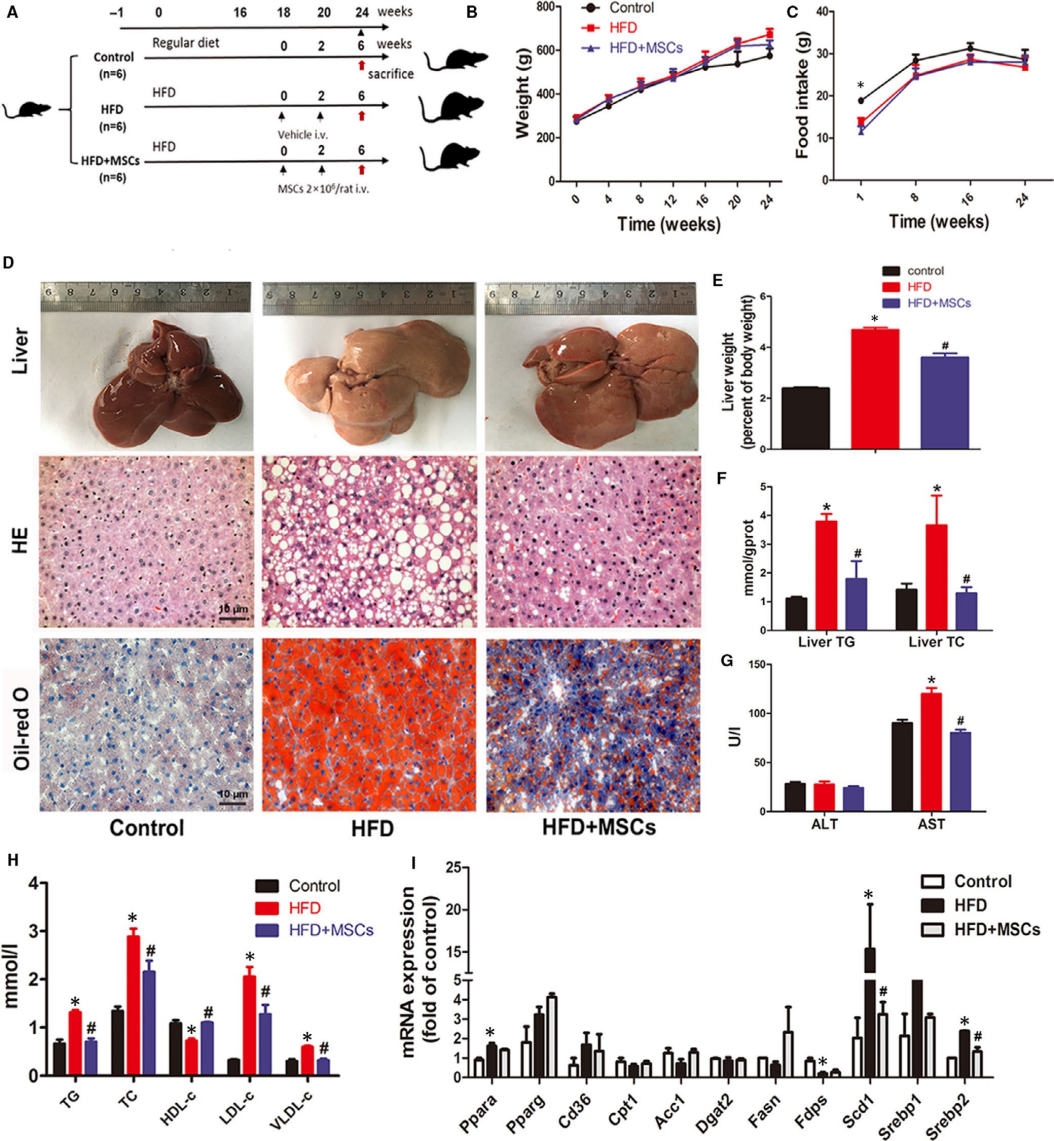
rMSC administration ameliorated HFD‐induced dyslipidaemia and steatosis in liver. Protocol for rMSC therapy in high‐fat diet–fed rats (A): After 18 wk of HFD feeding, SD rats were separated into two groups, one group received two doses of 2 × 10^6^ rMSCs (HFD + MSCs group, n = 6). The other group received the vehicle (HFD group, n = 6). Both groups were fed HFD during all study period (24 weeks). A third group of rats was fed exclusively with regular diet (control group, n = 6). Changes in bodyweight (B) and food intake (C) in three groups. Macroscopic image of liver (top), H&E staining (middle) and Oil Red O staining (bottom) of liver sections of the three groups at the 24th week (D). Liver/bodyweight ratio (E), levels of liver TG and liver TC (F), serum levels of AST, ALT (G) and lipid (H) in the three groups. And the mRNA levels of lipid metabolism‐relative genes in the liver tissues of the three groups (I), and the experiment was repeated 3 times independently. The data are expressed as mean ± SD, *P < .05 vs the control group; ^#^ *P* < .05 vs the HFD group

Eighteen male Sprague Dawley rats (6‐8 weeks) were obtained from the DASHUO animal company and housed in the Animal Center of West China Hospital, Sichuan University. Protocols for animal study were approved by the Institutional Animal Care and Use Committee (IACUC) of West China Hospital, Sichuan University. The rats were kept in standard laboratory conditions with free access to food and water and were allowed to adapt to the new environment for two weeks before experimental procedures. Rats were randomly assigned to a control group (fed with chow diet, n = 6), a HFD–fed group (2% cholesterol, 10% lard, 20% sucrose, 0.2% sodium cholate and 67% standard diet, n = 6) and a MSC‐treated group (HFD + MSCs, n = 6). The chow diet and HFD were purchased from Sichuan Academy of Agricultural Cultural Science. The energy contribution in percentual of nutrient content for each diet is listed in Table 1. After 18 weeks HFD feeding, rats of HFD + MSCs group were administered of MSCs (2 × 10^6^ cell/rat, suspended in 1 mL saline) via the rat's tail vein for two times with an interval of 2 weeks, whereas controls received 1mL saline. Four weeks after the last injection, all the animals were killed for sample collection (Figure 1A).

**TABLE 1:** The energy contribution in per cent of nutrient content for each diet

Diet	Total energy (Kcal/g)	Fat (%)	Cholesterol (%)	Protein (%)	Carbohydrate (%)
Chow	1.29	27.4	0	56.77	15.77
HFD	2.90	37.84	4.95	18.07	23.35

#### 2.1.2 Isolation and culture of rat bone marrow–derived MSCs

Rat MSCs (rMSCs) were harvested from the bone marrow of healthy SD rats (80‐100 g) and cultured as described previously.^12^ Briefly, the rats were killed by an intraperitoneal injection of pentobarbital sodium (30 mg/kg bodyweight), and bone marrow cells were aspirated from the femurs and tibias. Then, cells were plated in 25‐cm^2^ flasks with Dulbecco's modified Eagle's medium (HyClone) containing 15% foetal bovine serum and 1% penicillin‐streptomycin, at 37°C. After 72 h, the medium was replaced with fresh medium, and the adherent cells were defined as passage 0 MSCs. The surface markers of MSCs were identified by flow cytometric analysis using fluorophore‐conjugated antibodies: anti‐rat CD29‐FITC, CD44‐FITC, CD34‐PE and CD45‐PE (BD Biosciences, Franklin Lakes, NJ, USA). Cells at passages 3 or 4 were used for experiments.

#### 2.1.3 Physical and biochemical analyses

Bodyweight, food intake and fasting glucose level were recorded every month. Blood was collected from tail vein, and plasma was isolated by centrifugation. Serum aspartate aminotransferase (AST), alanine aminotransferase (ALT), triglyceride (TG), total cholesterol (TC), low‐density lipoprotein cholesterol (LDL‐C) and HDL‐cholesterol (HDL‐C) levels were measured with an auto‐analyser (Cobas 6000 c501; Roche Diagnostics). Fasting blood glucose was measured with a One‐Touch Accu‐Chek Glucometer (Roche Diagnostics), and fasting serum insulin was determined using a rat‐specific insulin ELISA kit (Ultrasensitive Rat Insulin ELISA; Mercodia) following the kit instruction.

#### 2.1.4 IPGTTs and IPITTs

Intraperitoneal glucose tolerance tests (IPGTTs) were carried out on 16‐h fasted rats, and blood glucose levels were determined with a One‐Touch Accu‐Chek Glucometer (Roche Diagnostics) at 0, 5, 10, 30, 60 and 120 min by venous tail bleeding after a 2 g/kg body weight injection of glucose solution (Kelun). Intraperitoneal insulin tolerance tests (IPITTs) were performed after 6‐h fasting, blood glucose levels were measured as described above at 0, 5, 15, 30, 60 and 90 min after a 1.0 U/kg intraperitoneal injection of insulin solution (Wanbang).

#### 2.1.5 Histological analysis

All the animals were killed by an intraperitoneal injection of pentobarbital sodium (30 mg/kg bodyweight) after 24 weeks, and tissue biopsies from liver and pancreas were performed. Liver samples were fixed overnight in 4% paraformaldehyde in phosphate buffer saline (PBS) at 4°C. Serial sections of the right lobes of the liver were stained with haematoxylin and eosin (H&E), periodic acid‐Schiff stain (PAS) and Masson's trichrome. Frozen sections were stained with Oil Red O to visualize lipid accumulation. Immunohistochemistry staining with an anti‐insulin antibody (Santa Cruz) was used to confirm the presence of β‐cells. Images were captured with a digital camera and analysed blindly by two observers.

#### 2.1.6 Liver TG and TC measurements

Liver total triglycerides and total cholesterol were determined with a Total Triglyceride kit and a total cholesterol kit (Nanjing Jiancheng Bioengineering Institute), respectively, according to the manufacturer's instructions. The lipid extraction was conducted as follows: 270 µl of 100% ethanol was added to 30 mg of liver tissue, and then, the tissue suspension was centrifuged at 1500 *g* for 10 min at 4°C. The supernatant was aspirated for subsequent test.

### 2.2 In vitro

#### 2.2.1 Cell culture and treatment

The human hepatocellular carcinoma cell line HepG2 cells (obtained from ATCC) were cultured in high‐glucose (25 mmol/L) Dulbecco's modified Eagle's medium (DMEM) supplemented with 10% (v/v) foetal bovine serum (FBS) (Wisent, Canada) and penicillin (100 U/mL) and streptomycin (100 mg/mL), at 37°C with 5% CO_2_. All the experiments were performed when the cells reached about 80% confluency, and the FBS was reduced to 2% when treatment began.

Human umbilical MSCs (hMSCs) were provided by Sichuan Neo‐Life Stem Cell Biotech Institution and identified by flow cytometry (Beckman Coulter) with specific anti‐CD31‐PE and anti‐CD144‐FITC antibodies (BD, PharmingenTM). Cells between passages 3 and 6 were used in all experiments reported herein.

For coculture experiments, hMSCs were seeded in a transwell insert at a density of 1.0 × 10^4^/cm^2^ and cultured in complete low‐glucose DMEM for 12 h before coculture with HepG2 cells. Prior to culture, hMSCs were washed with PBS three times and cocultured with HepG2 (ratio 1:5) in high‐glucose DMEM with 2% FBS, and the final PA concentration was consistent with HepG2 cells in all experiments.

#### 2.2.2 Cell viability assay

HepG2 were seeded in 96‐well plate and treated with indicated conditions. After treatment, 10 µl of CCK‐8 solution (Dojindo) was added to each well, and the plates were incubated for a further 2 h at 37°C. The absorbance at 450 nm was measured by microplate reader (BioTek Instruments Inc).

#### 2.2.3 Detection of intracellular lipid droplets (LD) and cellular triglycerides

HepG2 cells were exposed to palmitic acid or BSA as control for 24h. Cells were fixed in 4% paraformaldehyde in PBS for 30 min and stained with the working solution of Oil Red O for 1 h at 37℃ and imaged by microscope. For quantitative analysis of intracellular lipid droplets, the cells were exposure to 60% isopropanol for 30 seconds, and exhaustively rinsed with water. After evaporating the water, 200ul isopropyl alcohol was added and the extracted dye was monitored by a spectrophotometer at 510 nm. Cellular TG was determined by a Triglyceride Assay Kit (Sigma).

#### 2.2.4 ATP measurement

ATP content of HepG2 cells was measured by an ATP Assay Kit (Beyotime Biotechnology) according to the manufacturer's instructions. The ATP level was presented as nanomoles per milligram of protein.

#### 2.2.5 IL‐1β and IL‐18 measurement

Cell culture supernatants were used to measure the levels of cytokine IL‐1β (ab46052; abcam) and IL‐18 (ab215539; abcam), using ELISA kits according to the manufacturer's instructions.

#### 2.2.6 Detection of intracellular Ca^2+^ signalling and SERCA activity analysis

After treatments, the HepG2 cells were harvested and washed with HBSS. The cytosolic free calcium was labelled by 1 μmol/L Fluo‐4 AM (Invitrogen). Subsequently, the cells were washed with calcium‐free HBSS and then incubated in HBSS to allow complete esterification of the probe. A confocal microscope (Nikon Ti A1) was used to evaluate the free cytosolic Ca^2+^. Calcium release from the ER was initiated using 5 μmol/L thapsigargin, which inhibits Serca2b‐mediated calcium reuptake. Calcium release into cytosol was monitored as time‐dependent increase in Fluo‐4 AM fluorescence intensity at excitation and emission wavelengths of 494 and 516 nm detected by flow cytometry (Beckman, USA). The activity of SERCA in the HepG2 cells was determined using a Ca^2+^ ATPase activity assay kits (Genmed Scientifics) and normalized to the protein concentration.

#### 2.2.7 Quantitative real‐time PCR

Total RNA was extracted using TRIzol reagent (Gibco, Life Technologies) and reverse‐transcribed into cDNA using an iScript cDNA synthesis kit (Bio‐Rad). Real‐time polymerase chain reaction (real‐time PCR) was performed on CFX96 real‐time PCR detection system (Bio‐Rad) with SYBR Green (Bio‐Rad). Primers used in this study are listed in Table 2, commercial primers that are designed and validated by the Sango Biotechnology, and the relative change in mRNA expression was calculated by delta‐delta Ct method with β‐actin as reference gene.

**TABLE 2:** Primers for real‐time RT‐PCR

Gene	Sequence 5′‐3′	
	Forward	Reverse
*hACC1*	GCCTCCAACCTCAACCACTA	AAGGTCCGGAAAGAGACCAT
*hBIP*	AAGAACCAGCTCACCTCCAA	CACCTTGAACGGCAAGAACT
*hCASPASE‐1*	GATGATGATCACCGGTTTG	GAAGAAACACTCTGAGCAAGTC
*hCD36*	CTCGGATGGCTAGCTGATTACT	GCACTTGCTTCTTGCCAACT
*hCPT1*	GATGTGGACCTGCATTCCTT	TCCTTGTAATGTGCGAGCTG
*hDGAT2*	CGCAGCGAGAACAAGAATAA	TCTGTTGAGCCAGGTGACAG
*hEDEM1*	TACCAGGCAACCAAGAATCC	CCGGTCTTCTGTGGACTTGT
*hERP72*	GCCTACCAGCAATACCAGGA	CTCAGGCTGCATTACAACCA
*hFASN*	ATGCACACAGTGCTCAAAGG	TGAGAACAGGCTCTCCCACT
*hFDPS*	TCTGACGGTGGTACAGACCT	ACACGAGGAAGAAAGCCTGG
*hFKBP11*	CCTTCTCACTTGGCCTATGG	TTGGCTCGGATTAGTGCAA
*hGRP90*	AGCCTCTGCTGAATTGGATG	GTTGCCAGACCATCCGTACT
*hGSDMD*	GCCAGAAGAAGACGGTCACCATC	TTCGCTCGTGGAACGCTTGTG
*hIL18*	TGATATCGACCGAACAGCCAACG	GGTCACAGCCAGTCCTCTTACTTC
*hIL1b*	GCCAGTGAAATGATGGCTTATT	AGGAGCACTTCATCTGTTTAGG
*hNLRP3*	GGCAAATTCGAAAAGGGGTATT	CTGATTTGCTGAGAGATCTTGC
*hPPARa*	ACCTGAGGAAGCCATTCTGC	GTTTAGAAGGCCAGGCCGAT
*hPPARg*	TGAACGTGAAGCCCATCGAG	ATCTTCTGGAGCACCTTGGC
*hSCD1*	CGTGGCTTCTTGTGGTGTTG	ATACCAGGAGGAATGGGCCT
*hSERCA2b*	GTGAACGATGCTCCTGCTCTGAAG	TGGACGAGATGAGGTAGCGGATG
*hSREBP1*	CCAGCCTTTGAGGATAACCA	TGCAGGTCAGACACAGGAAG
*hSREBP2*	GCAACAACAGCAGTGGCAGAG	TGAGGGAGAGAAGGTAGACAATGG
*hTRIB3*	AGGAAGAAGCGGTTGGAGTT	TGCACGATCTGGAGCAGTAG
*hACTIN*	CCACGAAACTACCTTCAACTCC	GTGATCTCCTTCTGCATCCTGT
*rAcc1*	GCCTCCAACCTCAACCACTA	AAGGTCCGGAAAGAGACCAT
*rBip*	CAAGAACCAACTCACGTCCA	AACCACCTTGAATGGCAAGA
*rCaspase‐1*	AGAGTCTGGAGCTGTGGCTACTG	ATGAGTGCTTGCCTGTGTTGGTC
*rCd36*	CTCGGATGGCTAGCTGATTACT	GCACTTGCTTCTTGCCAACT
*rCpt1*	GATGTGGACCTGCATTCCTT	TCCTTGTAATGTGCGAGCTG
*rDgat2*	CGCAGCGAGAACAAGAATAA	TCTGTTGAGCCAGGTGACAG
*rEdem1*	GGAAATTCATCCGAGTTCCA	GAAAGGAGGCTTCCCAGAAC
*rErp72*	AGCAGCTGGAGCCTGTCTAC	CTCCACCTTGTATCGGTCGT
*rFasn*	ATGCACACAGTGCTCAAAGG	TGAGAACAGGCTCTCCCACT
*rFdps*	TCTGACGGTGGTACAGACCT	ACACGAGGAAGAAAGCCTGG
*rFkbp11*	TACACTACACGGGCAGCTTG	CAGAAGGCTCTGCTCCAGAC
*rFn*	*GACACTATGCGGGTCACTTG*	*CCCAGGCAGGAGATTTGTTA*
*rGrp94*	CTGATGATGAAGCCGCAGTA	GCCTCTGCCATATTGGTTTG
*rGsdmd*	GTCTGCTTGCCGTACTCCATTCC	TGAAGAGCCTGCCTCCACCTC
*rIl‐18*	TGATAATGCTAGCGAACAGCCAACG	GGTCACCTAGTTAGTCCTCTTACTTC
*rIl1b*	GCCAACAAGTGGTATTCTCCA	TGCCGTCTTTCATCACACAG
*rNlrp3*	GAGCTGGACCTCAGTGACAATGC	ACCAATGCGAGATCCTGACAACAC
*rPpar a*	ACCTGAGGAAGCCATTCTGC	GTTTAGAAGGCCAGGCCGAT
*rPpar g*	TGAACGTGAAGCCCATCGAG	ATCTTCTGGAGCACCTTGGC
*rScd1*	CGTGGCTTCTTGTGGTGTTG	ATACCAGGAGGAATGGGCCT
*rSerca2b*	AGTTCATCCGCTACCTCATCTCCTC	GCAGACCATCCGTCACCAGATTG
*rSrebp1*	CCAGCCTTTGAGGATAACCA	TGCAGGTCAGACACAGGAAG
*rSrebp2*	GCAACAACAGCAGTGGCAGAG	TGAGGGAGAGAAGGTAGACAATGG
*rTgfb*	AATTCCTGGCGTTACCTTGG	TCTCCTTGGTTCAGCCACTG
*rTnfa*	GCTCCCTCTCATCAGTTCCA	GCTTGGTGGTTTGCTACGAC
*rTrib3*	AGCAAGGCAACTGCACCTAT	GTGGGTAGGCAGTCTTGCAT
*rActin*	CTGGGACAGCAGCCTGTATT	CGCTAGCCCTTGACTCTCTC

#### 2.2.8 siRNA transfection

HepG2 cells were transfected with a specific SERCA2b (ATP2A2) siRNA (100 nmol/L) or non‐binding control siRNA (100 nmol/L) using Lipofectamine 2000 (Invitrogen) according to the manufacturer's instructions. The sequences of SERCA2b siRNA were as follows: sense 5′‐GCCUGGAACAGGUCAAGAATT‐3′ and antisense 5′‐UUCUUGACCUGUUCCAGGCTT‐3′ (GenePharma). After transfection for 24 h, the cells were exposed to PA with or without MSC coculture for a further 24 h.

#### 2.2.9 Western Blot analysis

Frozen liver tissues and cultured hepatocytes were lysed in ice‐cold RIPA buffer supplemented with a cocktail of protease and phosphatase inhibitors (Calbiochem) and then centrifuged at 7200 *g* for 15 min at 4°C. The protein concentration was determined by BCA Protein Assay Kit (CWBIO). Equal amounts of protein were separated on SDS‐PAGE and transferred to PVDF membrane (Millipore). After blocked with 5% bovine serum albumin or non‐fat milk for 1 h at room temperature, the membranes were incubated overnight at 4°C with primary antibodies against p‐eIF2α (Ser51) and eIF2α (WL01909, Wanlei); ATP2A2/SERCA2, phosphorylated protein kinase B (p‐Akt) and Bax (3398, 4388, 9271, 2772; Cell Signaling Technology); ATF‐6 and CHOP (DF6009, DF6025; Affinity); Grp78(Bip) (abs130538a; Absin); p‐IRS (Tyr896) (ab46800; Abcam); IRS‐1 (WL03123; Wanlei); BCL‐2 (A2212; Abclonal); SREBP‐1c (sc‐13551; Santa); Xbp1s and ATF‐4 (ET1703‐23, ET1612‐37; HUAbio); NLRP3 (DF7438; Affinity); gasdermin D (GSDMD) and gasdermin‐N domain (GSDMD‐N) (207701‐AP; Proteintech); pro‐caspase‐1 (WL02996; Wanlei); cleaved caspase‐1 (p20) (WL02996a; Wanlei); mature IL‐1 beta (AF4006; Affinity); and mature IL‐18(bs‐4988R‐1; Bioss). The antibody against β‐ACTIN (AC006; Abclonal) was used as a loading control. The immunoblots were visualized using a ChemiDoc^TM^ imaging system (Bio‐Rad, USA) and quantified by the ImageJ software.

#### 2.2.10 Statistical analysis

Experiments were performed at least three times, and quantitative data are expressed as mean ± SD All data were analysed using the SPSS software (version 17.0), and comparison between two groups of values was performed by t test. A value of *P* < .05 was considered to be at significant difference.

## 3 RESULTS

### 3.1 rMSC therapy improved lipid metabolism in HFD‐fed rats

The bodyweights and food intake of the HFD group showed no significant difference in comparison with the control group after 24 weeks HFD (Figure 1B,C). However, rMSC treatments significantly attenuated the hyperlipidaemia of HFD‐fed rats (Figure 1D‐H). In respect of the lipid accumulation in liver, HFD has obviously developed liver steatosis at the end of experiment duration, and systemic rMSC transplantation prevented the development of HFD‐induced fatty liver, showing substantially decreased macrovesicular steatosis and lipid droplet accumulation in hepatocytes in H&E and Oil Red O staining of liver sections (Figure 1D), as well as decreased liver weight/bodyweight ratio and liver TG and TC contents (Figure 1E,F). Additionally, the serum levels of TG, TC and LDL‐c were increased in HFD rats but decreased in rMSC‐treated rats, while the HDL‐c level was decreased in HFD rats but increased in rMSC‐treated rats (Figure 1H). And AST was increased in HFD rats but decreased in rMSC‐treated rats, while the ALT levels showed no significant changes in the three groups (Figure 1G), suggesting that rMSCs have potential to improve liver functional abnormality.

Furthermore, the mRNA levels of lipid metabolism‐related genes in the liver were determined by qPCR. The results suggested that the expressions of many metabolic genes were changed in the HFD group at the 24^th^ week, including FFA transport‐ and synthesis‐related genes such as *Ppara and Scd1*. Particularly, the gene expressions of *Srebp1* and *Srebp2*, which are activated by the ER stress pathway^7^, ^13^, ^14^ and known as key regulators of lipid and cholesterol synthesis, respectively, were promoted in the HFD rats but inhibited in rMSC‐treated rats (Figure 1I).

### 3.2 rMSCs improved insulin sensitivity in HFD‐fed rats

**FIGURE 2:**
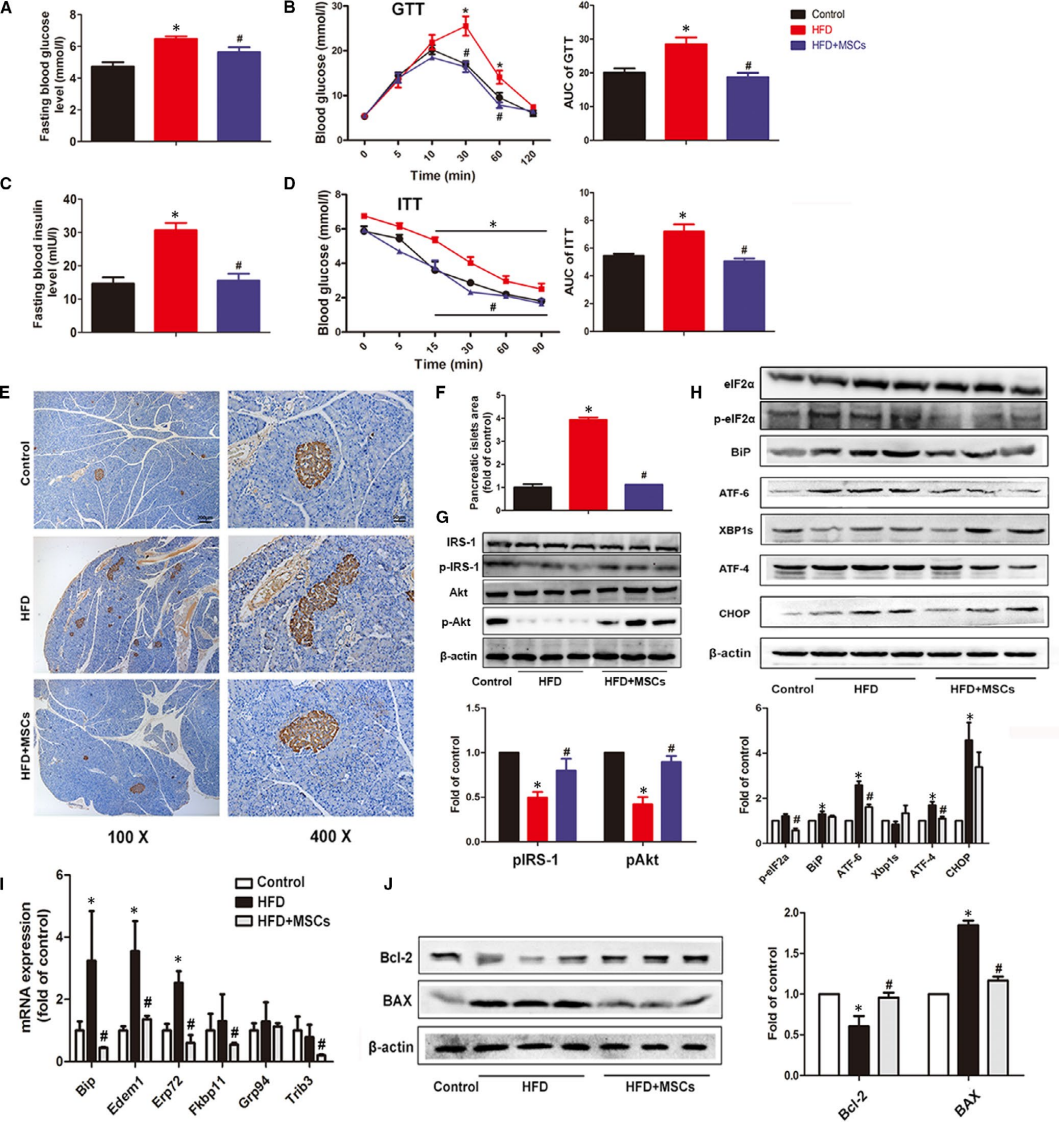
Effects of rMSC administration on glucose metabolism, insulin pathway and liver ER stress of HFD‐fed rats. Fasting blood glucose and insulin levels of the three groups were measured at the 24th week (A, C). Blood glucose levels in a glucose tolerance test (GTT) and an insulin tolerance test (ITT) (B, D). Representative images of pancreas stained with anti‐insulin antibody in the three groups (E) and the quantitative analysis of the insulin‐positive areas in the three groups (F). The protein levels of p‐IRS‐1 and p‐Akt in the liver tissues from the three groups were measured by Western blotting, β‐actin was used as a protein loading control (G). The protein levels of ER stress markers in liver were changed in the three groups at the 24th week (H); the mRNA expression of ER stress–relative genes in liver of the three groups (I); the Bcl‐2 and BAX expressions were measured by Western blotting, and β‐actin was used as protein loading control (J), and the experiment was repeated 3 times independently. The data are expressed as mean ± SD, *P < .05 vs the control group; ^#^ *P* < .05 vs the HFD group

To explore the effects of rMSC treatment on glucose metabolism of HFD‐fed rats, we measured the fasting glucose levels and serum insulin levels at the 24^th^ week. The fasting glucose levels were slightly increased in HFD‐fed rats but decreased in rMSC‐treated rats (*P* > .05). The fasting serum insulin levels were remarkably increased in HFD‐fed rats indicating a status of hyperinsulinaemia, while rMSC treatments reversed the insulin to normal levels (Figure 2A,C). To further confirm the development of insulin resistance in HFD rats, we did IPGTT and IPITT tests, and analysed the major proteins involved in insulin signalling pathway. rMSCs improved the insulin sensitivity in the HFD‐fed rats indicated by the reduced area under the curve (AUC) of IPGTT and IPITT analysis (Figure 2B,D). Meanwhile, immunohistochemical staining for rat insulin in the pancreatic tissue of the HFD rats showed more insulin‐positive β‐cell mass in comparison with the control group, implying a compensatory hyperplasia process of pancreatic islets in the context of peripheral insulin resistance (Figure 2E,F). In addition, the phosphorylated protein levels of insulin signalling including p‐IRS‐1 (Tyr896) and p‐Akt (Ser473) were decreased in the HFD group but were restored in the rMSC‐treated rats (Figure 2G). These results demonstrated that rMSCs restored systemic and hepatic glucose homeostasis in HFD‐fed rats.

### 3.3 rMSCs inhibited ER stress in the liver of HFD‐fed rats

After 24 weeks, HFD strongly induced hepatic ER stress, as indicated by increased protein levels of PERK and ATF6 pathway markers including p‐eIF2a, ATF4 and ATF6, as well as the chaperone protein BiP. But the sliced XBP did not show significant difference. However, these up‐regulations were significantly inhibited by rat MSCs treatment (Figure 2H). Consistently, the mRNA expressions of UPR‐related genes including *Bip*, *Edem1* and *Erp72* were increased after HFD, but decreased in the rMSC‐treated group (Figure 2I). Apoptosis is believed to be closely linked to ER stress. As we expected, the protein expression of pro‐apoptotic molecule BAX was markedly increased in the HFD group but decreased in the rMSC‐treated group. Conversely, the antiapoptotic molecule Bcl‐2 was increased in the rMSC‐treated group compared with its decrease in the HFD group (Figure 2J).

### 3.4 In vitro

#### 3.4.1 hMSCs ameliorated PA driving hepatic lipotoxicity in HepG2 cells

**FIGURE 3:**
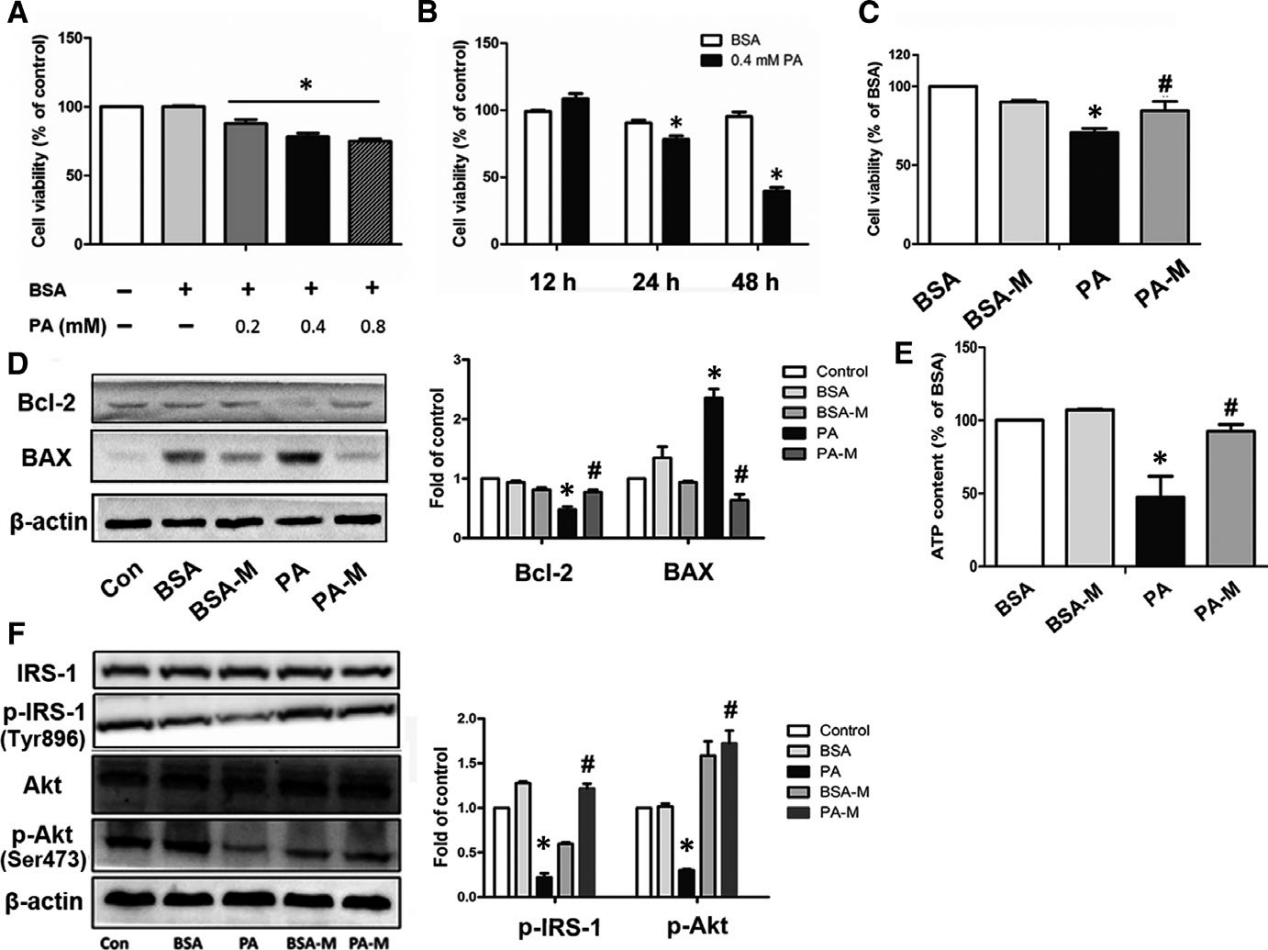
hMSCs ameliorated PA‐induced injury in HepG2 cells. Time‐ and dose‐dependent effects of PA on HepG2 cells (A, B). HepG2 cells were incubated with 0.4 mmol/L BSA or PA and cocultured with or without hMSCs, and the cell viability (C), Bcl‐1 and BAX protein levels (D), ATP content (E) and insulin pathway–relative proteins levels (F) were measured after 24 h. BSA‐M represents BSA + MSCs, and PA‐M represents PA + MSCs. Results are presented as means ± SD from three independent experiments. *P < .05 vs the control or BSA group; ^#^ *P* < .05 vs the PA group

In agreement with previous reports,^15^, ^16^ we found that palmitic acid (PA) deteriorated cell viability in a time‐ and dose‐dependent manner in HepG2 cells. Cell viability levels were decreased with the increase in PA concentration after 24h, and when HepG2 cells were cultured with 0.4 mmol/L PA for 24 h, the cell viability was decreased to 67.58% of that of the BSA control, but hMSCs coculture substantially restored the cell viability to 84.61% (Figure 3A‐C). Additionally, the PA‐induced promotion of BAX expression and inhibition of Bcl‐2 expression were reversed by hMSC addition, implying MSCs had potential to attenuate PA driving cell apoptosis (Figure 3D). As a long‐chain SFA, PA is β‐oxidized in mitochondria, and PA‐induced lipotoxicity is able to cause mitochondrial dysfunction, which is indicated by impaired bioenergetics.^17^ So, we determined the ATP production after the cells were cultured with PA for 24 h. The results showed that the ATP levels of HepG2 cells in the PA‐treated group were decreased but were restored in the PA‐MSC group (Figure 3E). In addition, the p‐Akt (Ser473) and p‐IRS‐1 (Try896) protein levels of HepG2 were decreased in the PA group but increased in the PA‐M group (Figure 3F).

Additionally, we also demonstrated rMSCs alleviated PA‐induced lipotoxicity in primary rat hepatocytes ([Link], [Link]), as shown in Figure S1.

#### 3.4.2 Effects of hMSCs on lipid metabolism in PA‐treated HepG2 cells

**FIGURE 4:**
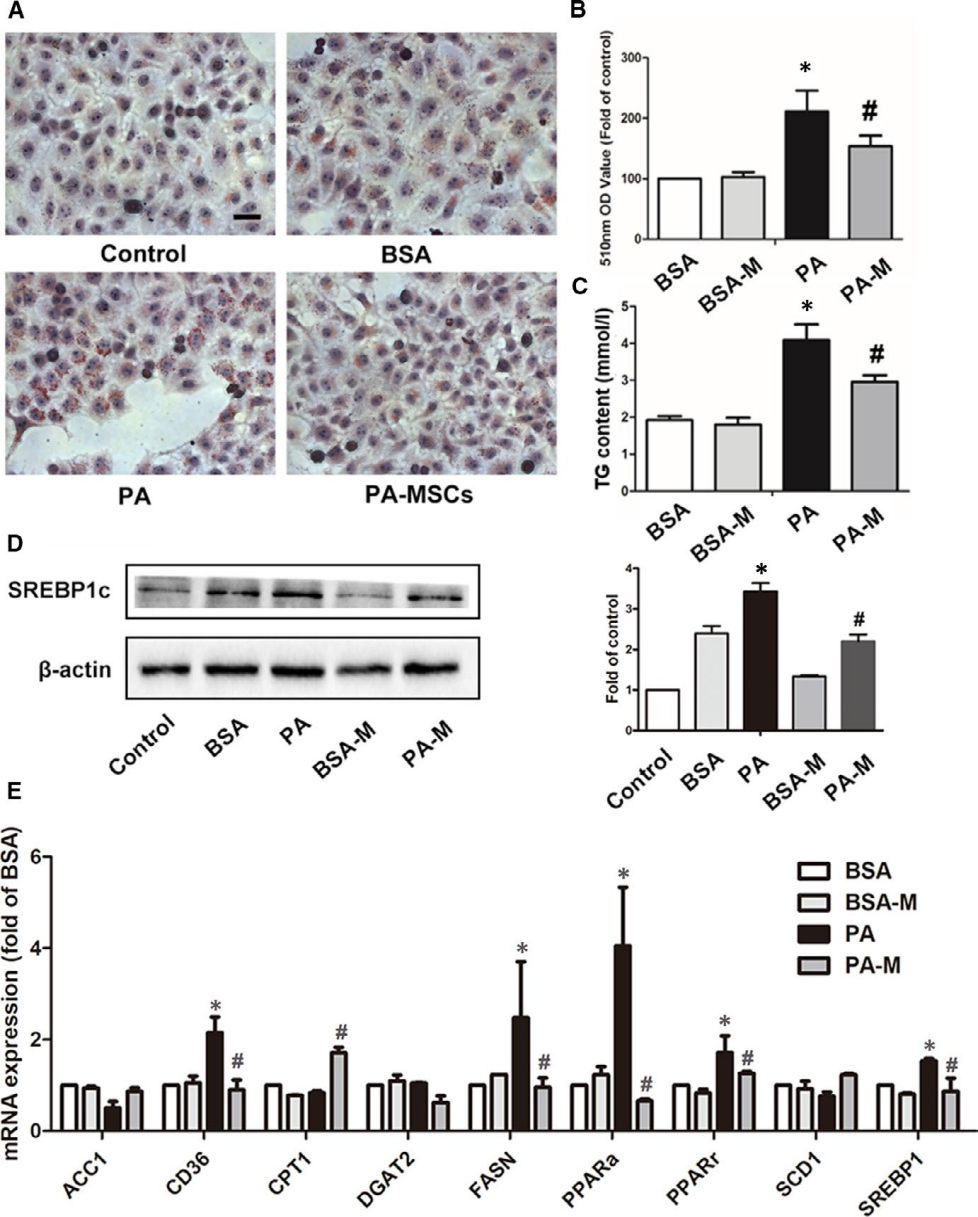
hMSCs alleviated PA‐induced hepatic steatosis in HepG2 cells. Cells were stained with Oil Red O, and lipid accumulation was visualized under a microscope at 400 × magnification after 24‐h treatment (A, B). The cellular TG levels in the three groups (C). The protein levels of SREBP‐1c were measured by Western blotting, and β‐actin was used as an internal control (D). The mRNA expression levels of genes governing lipid metabolism were detected after 12‐h treatment (E). Results are presented as means ± SD from three independent experiments, *P < .05 vs the control or BSA group; ^#^ *P* < .05 vs the PA group

After HepG2 cells were exposed to 0.4 mmol/L PA for 24 h, cellular lipid accumulation was determined by Oil Red O stain. The results showed that very few lipid deposits were observed in the control or BSA groups, while massive droplets were present in the cytoplasm of PA‐treated cells. Interestingly, MSC coculture obviously inhibited the accumulation of lipid droplets after PA stimulation (Figure 4A,B). Consistently, PA treatment for 24 h nearly doubled the TG content of HepG2 cells compared with that of BSA control cells, while cocultured with hMSCs for 24 h showed an elevated TG level compared with that of controls but a reduced level compared with that of the PA group (Figure 4C). Additionally, the protein level of SREBP1c (the cleaved fragment of SREBP1), which is the master transcription factor that control lipogenesis and lipid uptake, was significantly increased in the PA group but dropped down in the PA‐M group (Figure 4D). These results suggested that hMSCs protected the HepG2 cells from PA‐induced TG accumulation.

The mRNA expression of lipid metabolism‐related genes was significantly altered after culture with PA for 12 h. Interestingly, the expressions of fatty acid transport‐related genes *CD36* and lipid synthesis genes including *PPARalpha*, *PPARgamma, FASN* and *SREBP1c* were up‐regulated in the PA group but decreased in the PA‐M group. In addition, the PA‐M group showed an increase of mRNA level of *CPT1A*, a mitochondrial enzyme responsible for the transfer of fatty acids to mitochondria for β‐oxidation (Figure 4E).

#### 3.4.3 hMSCs alleviate PA‐induced ER stress in hepatocytes

**FIGURE 5:**
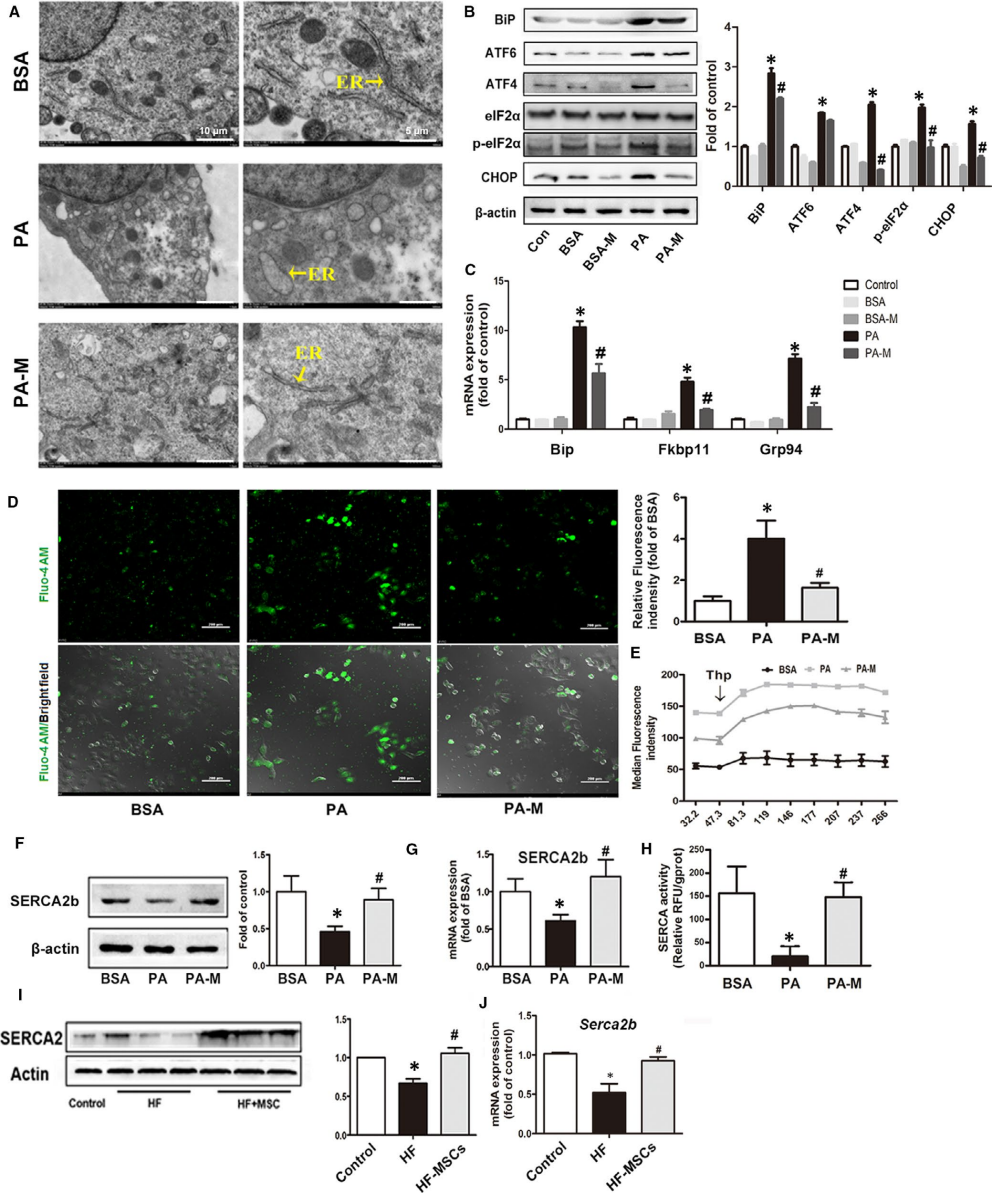
MSCs alleviated PA‐induced ER stress in HepG2 cells. The morphology of the ER in the HepG2 cells was observed by electron microscopy (A). The mRNA expression and proteins levels of ER stress makers and the protein levels of BiP, ATF6/4, p‐eIF2α and CHOP in HepG2 cells were measured after 24 h (B, C). Representative Fluo‐4AM ratio images of cytosolic calcium in HepG2 cells after 24 h, and quantification of the relative fluorescence intensity (D). The intracellular calcium release was detected by flow cytometry (E). The protein and mRNA expression levels of SERCA in HepG2 cells after PA exposure for 24 h with or without MSC coculture (F, G). Measurement of the SERCA activity in HepG2 cells at 24 h after exposure (H). The protein and mRNA expression levels of SERCA in the livers of rats (I, J). Results are presented as means ± SD from three independent experiments, *P < .05 vs the control or BSA group; ^#^ *P* < .05 vs the PA or HFD group

Our previous studies have indicated that ER stress played a key role in SFA‐induced lipotoxicity both in hepatocyte and in pancreatic β cell.^18^, ^19^ To convince the effect of hMSC treatment on ER stress in HepG2 cells, we first observed the morphology of the ER using electron microscopy. The results showed that exposure to PA for 24 h induced marked ultrastructure abnormality in HepG2 cells. In respect of ER, the cisternae dilation and ribosome dropdown were the predominant alterations in the PA group. However, in the PA‐M group, these abnormal morphological changes in the ER were barely detected (Figure 5A). Additionally, PA exposure changed the expression of several typical ER stress marker proteins in HepG2 cells. The BiP, ATF‐6, ATF4, p‐eIF2a and CHOP were significantly increased in the PA group, while the hMSCs significantly decrease BiP, ATF‐4, p‐eIF2a and CHOP expression. In addition, the expression levels of UPR pathway‐related genes, including *GRP78*, *FKBP11* and *GRP94*, were up‐regulated in the PA group but down‐regulated in the PA‐M group (Figure 5B,C).

Then, we used Fluo‐4AM, a ratiometric calcium indicator dye, to investigate the alterations in intracellular calcium homeostasis. PA exposure for 24 h increased the concentration of resting cytosolic calcium in HepG2 cells, suggesting the calcium exflux from damaged ER calcium pool to the cytosol, or the defective calcium transport from the cytosol to the ER, while the hMSC treatment improved the PA‐induced calcium disequilibrium during ER stress and minimized the calcium overload in the cytosol (Figure 5D). To evaluate whether the elevation in cytosolic calcium was associated with the depletion in calcium storage in the ER, we utilized Thapsigargin (Thp), a specific inhibitor of SERCA, to induce depletion in the ER calcium stores. We detected the changes of cytosolic calcium levels during 300 s after Thp stimulation. The results showed that the cytosolic calcium levels in the PA group were approximately twofold higher than that of the BSA group before Thp stimulation, while, after Thp stimulation, the cytosolic calcium level showed much more increase in the PA group, implying that the PA exposure did not damage the calcium storage of ER, and the overload of cytosol calcium may due to the defective calcium transport from cytosol to the ER, rather than the exflux from ER. However, the addition of MSCs notably diminished perturbation of calcium homeostasis (Figure 5E).

Due to the critical role of the SERCA in the maintenance of calcium equilibrium in the ER, we measured the expression level and tested the activity of SERCA to investigate whether MSCs have a potential protection towards SERCAs. The results showed that PA exposure for 24 h decreased SERCA2 protein levels and SERCA2b mRNA levels and led to a severe impairment of SERCA activity in HepG2 cells. hMSC treatment rescued the SERCA activity and increased the protein and mRNA levels in HepG2 cells (Figure 5F‐H). We further convinced that in vivo, the SERCA expression levels in the liver of the HFD‐fed rats were significantly decreased but were increased in the HFD‐MSC group (Figure 5I,J).

#### 3.4.4 MSCs alleviate Thp‐induced hepatocyte injury and ER stress

**FIGURE 6:**
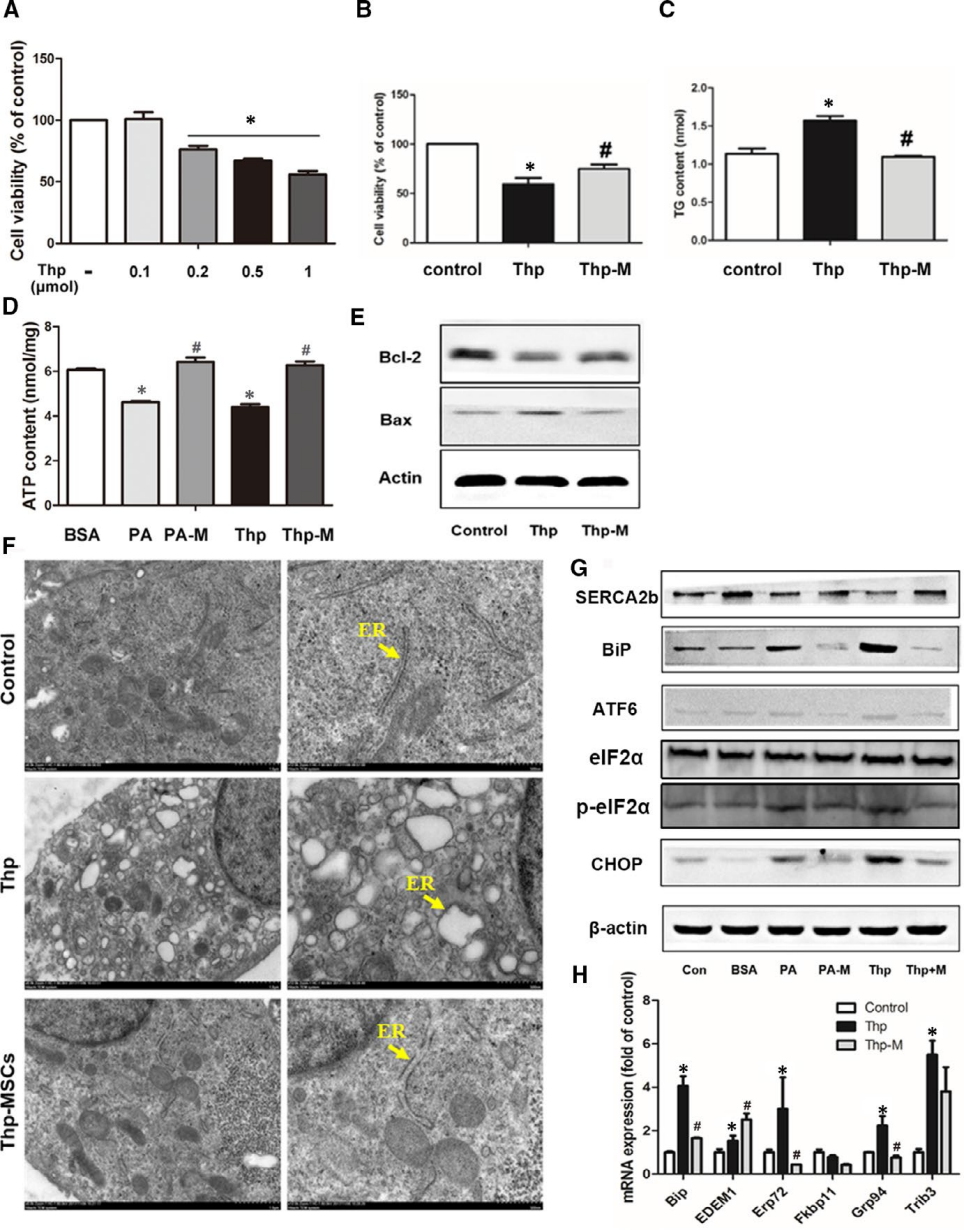
hMSCs alleviated the Thapsigargin (Thp)‐induced hepatocyte injuries and ER stress. Thp was utilized as a specific non‐competitive inhibitor of SERCA and showed dose‐dependent effects on HepG2 cells (A). The cell viability (B), intracellular TG content (C) and ATP content (D) of HepG2 cells were measured after 24 h exposure to 0.5 µmol/L Thp with or without hMSC coculture. Protein levels of Bcl‐2 and BAX in the liver tissues of the three groups were determined by Western blotting, and β‐actin was used as a loading control (E). The morphology of the ER in three groups was observed by electron microscopy (F). The proteins levels of ER stress markers and SERCA were measured by Western blotting (G), and the mRNA expression levels of ER stress markers were detected by qPCR (H) after 24 h. PA‐M represents PA + MSCs, and Thp‐M represents Thp + MSCs. Results are presented as means ± SD from three independent experiments, *P < .05 vs the control or BSA group; ^#^ *P* < .05 vs the PA or Thp group

To further convince the relevance of SERCA to MSC‐mediated protection against PA‐induced lipotoxicity in HepG2 cells, we utilized Thp as a specific non‐competitive inhibitor of SERCA and explored whether the MSC treatment could prevent the Thp‐induced ER stress and hepatocyte injury. We found that the cell viability was reduced to 60% of that of the control after 24 h of 0.5 µmol/L Thp treatment. However, the cell viability was restored to 80% in the MSC group (Thp‐M) (Figure 6A,B). Furthermore, the TG level of HepG2 cells in the Thp group was increased, confirming that ER stress is able to induce dysfunction of lipid metabolism, but it was decreased in the Thp‐M group (Figure 6C). Similar to the PA’s effects, the Thp‐treated HepG2 cells showed a deficiency in ATP production, which were improved in the Thp‐M group (Figure 6D). We also detected the expression of apoptosis‐related factors in Thp‐treated HepG2 cells, and as expected, the coculture of MSCs restored Bcl‐2 expression and inhibited BAX expression (Figure 6E). Additionally, similar to PA treatment, Thp induced marked ER abnormality in HepG2 cells, including the dilation of ER cisternae and ribosome dropdown through electronic microscopy. However, these morphological changes in the ER were scarcely detected in the Thp‐M group (Figure 6F). Furthermore, as an ER stressor, Thp exposure was observed to increase the protein levels of ER markers including BiP, p‐eIF2a‐ATF6 and CHOP in parallel with the activation of UPR pathway–related genes in HepG2 cells. Consistent with the results of the PA‐M group, MSC treatment alleviated the Thp‐induced ER stress (Figure 6G, H).

#### 3.4.5 SERCA plays an important role in MSC alleviation of PA‐induced abnormal metabolism in HepG2 cells

**FIGURE 7:**
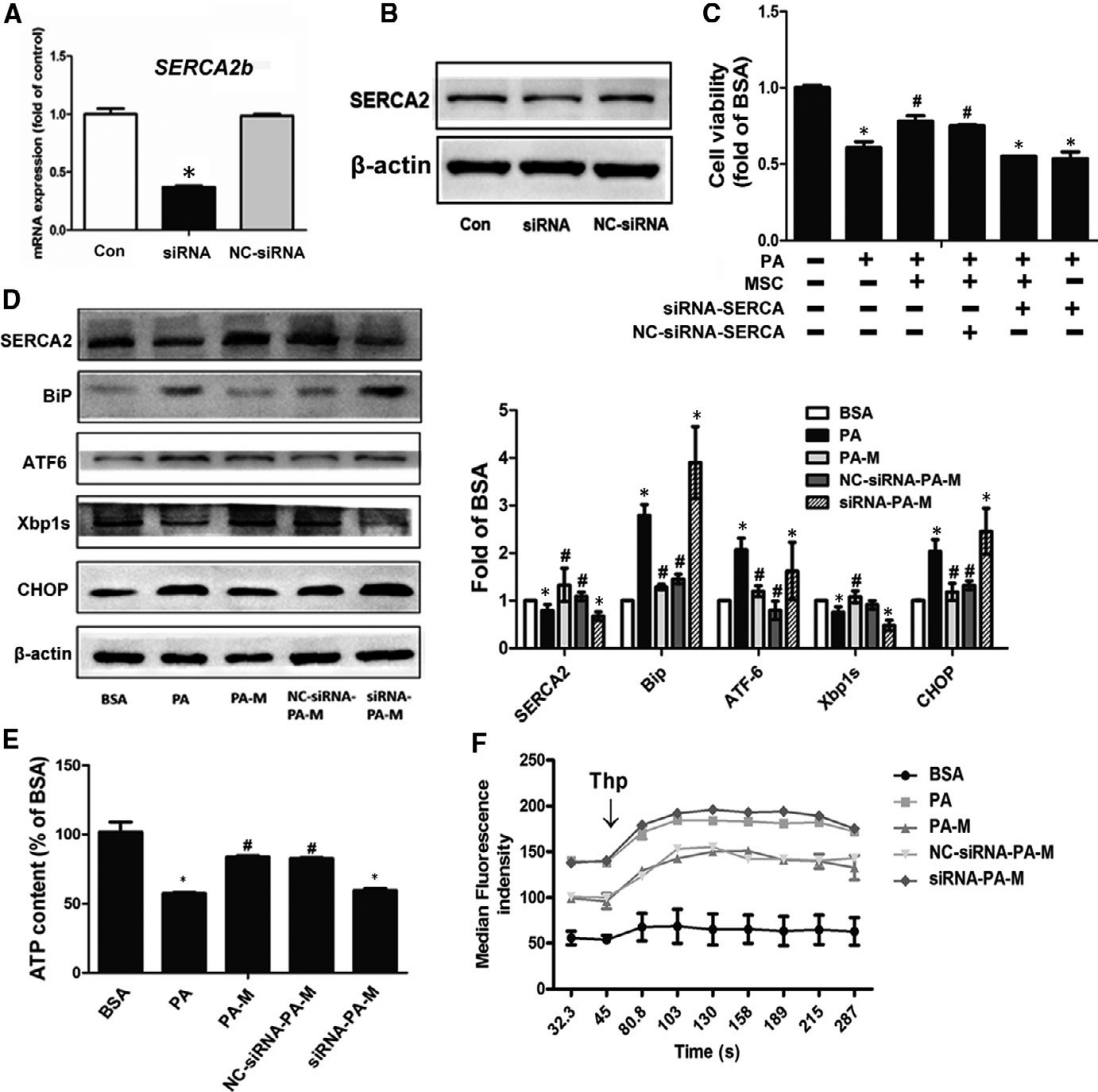
*SERCA2* silencing abolished the ability of hMSCs to reverse the PA‐induced lipotoxicity in HepG2 cells. Representative expression of mRNA and protein levels of SERCA2b after transfection (A, B). The cell viability of HepG2 cells after treatment (C). The proteins expression levels of ER stress markers and SERCA in different groups (D). The ATP content levels in different groups (E). The changes in cytosolic calcium levels after Thp stimulation at 300 s in all groups (F). Results are presented as means ± SD from three independent experiments. *P < .05 vs the control or BSA group; ^#^ *P* < .05 vs the PA group

We further explored whether the positive outcomes of MSC treatment were dependent on the improvement of SERCA function. We knocked down SERCA2b in HepG2 cells by *SERCA2b* siRNA transfection. The interference decreased 60% of the mRNA expression of *Serca2b*, in parallel with the decreased SERCA2 protein levels in the *SERCA2b* ^KD^ HepG2 cells. We cocultured *SERCA2b* ^KD^ HepG2 cells with MSCs and found that the MSC‐mediated protective effects on PA‐induced hepatocyte lipotoxicity were significantly diminished. As shown previously, MSCs improved the cell viability, ER stress and ATP content in PA‐induced HepG2 cells. However, all the benefits above were greatly abolished in the siRNA‐PA‐M group (Figure 7A‐E). Additionally, as expected, the genetic deficiency of SERCA blunted the regulatory effect of MSCs on intercellular calcium balance (Figure 7F).

#### 3.4.6 MSCs ameliorated PA‐induced cell death through inhibiting pyroptosis

**FIGURE 8:**
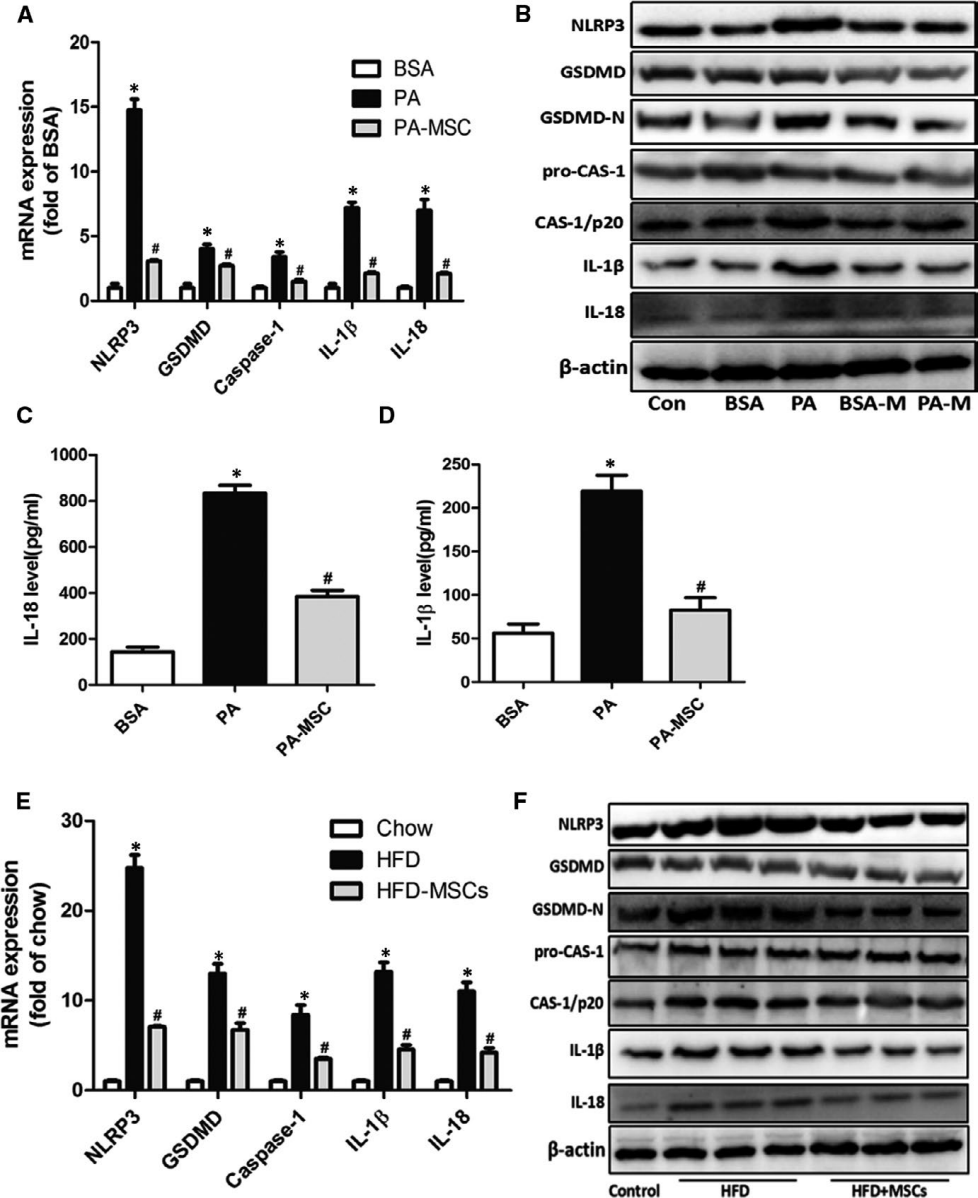
MSCs ameliorated hepatic cell death through inhibiting pyroptosis. The mRNA and protein levels of pyroptosis makers in HepG2 cells were measured after 24‐h treatment of PA (A, B). IL‐18 and IL‐1β in culture supernatant were measured by ELISA (C, D). The mRNA and proteins levels of pyroptosis markers in liver were detected in the three groups at the 24th week after HFD (E, F). Results are presented as means ± SD from three independent experiments, *P < .05 vs the control or BSA group; ^#^ *P* < .05 vs the PA or HFD group

It is well known that ER stress is a causal factor responsible for lipotoxicity‐induced cell death. Pyroptosis, a novel programmed cell death, is characterized by cellular lysis and release of pro‐inflammatory cytokines, and has been demonstrated to play a critical role in the progression of NASH.^20^ Here, we further explored the foremost pathway in PA‐induced cell death, focusing on pyroptosis, as well as the protective effect of MSCs. We examined the representative molecular markers of pyroptosis both in vivo and in vitro. The qPCR results showed that PA significantly up‐regulated the mRNA expression of Nlrp3, the typical marker of inflammasome after 24 h treatment, which paralleled the up‐regulations of Gsdmd, caspase‐1, IL‐1β and IL‐18 upon pyroptosis. Nevertheless, MSCs substantially attenuated the effects of PA on inflammasome and pyroptosis in HepG2 cells (Figure 8A). Consistently, the Western blot results indicated that PA substantially increased the protein levels of pyroptosis markers. The most distinct up‐regulations presented on NLRP3, GSDMD/−N (the activated form of GSDMD), caspase‐1, p20 (the activated form of caspase‐1), IL‐1β and IL‐18. As expected, these alterations were substantially abolished by MSC supplement (Figure 8B). Essentially, to convince the activation of pyroptosis pathway, we detected the release of mature IL‐1β and IL‐18 (Figure 8C,D). Consistently, ELISA confirmed that PA profoundly provoked the release of IL‐1β in HepG2 cells. However, MSCs were able to ablate this process. Furthermore, we investigated the effects of MSC treatment on liver pyroptosis in NAFLD rats. We found that long‐term HFD significantly facilitated the gene and protein expressions of pyroptosis markers including NLRP3, GSDMD/−N, caspase‐1, p20, IL‐1β and IL‐18, suggesting that pyroptosis is an important pathological process of NAFLD (Figure 8E,F). Notably, the MSC treatment group displayed alleviated pyroptosis, and the most distinct down‐regulations of pyroptosis markers presented on NLRP3, GSDMD/−N, caspase‐1, p20, IL‐1β and IL‐18, which confirmed the findings in HepG2 cells. Overall, these results revealed that long‐term HFD or PA stimulation would activate pyroptosis in hepatocytes, which may contribute to the cell death and liver dysfunction during the process of NAFLD, MSC treatment effectively ameliorates these deleterious effects, implying its potential to alleviate HFD‐induced pyroptosis in NAFLD.

## 4 DISCUSSION

MSCs have been shown to have beneficial effects in a wide spectrum of clinical settings, and accumulating evidence suggested that MSC therapy has potential to prevent HFD‐induced fatty liver development and the onset of NASH in mice.^21^ Kanna Nagaishi et al reported the rat bone marrow–derived MSC administration showed the curative effects on different pathology in liver of HFD–fed obese mice and STZ‐induced diabetic mice. The interesting point was that even though the hyperglycaemia has not been alleviated, diabetes‐induced liver dysfunction was remarkably reversed by MSC therapy,^22^ which suggested the MSCs may have more beneficial impact on lipid metabolism. Nevertheless, the exact mechanism of MSC effects on fatty liver and systemic dyslipidaemia has not been fully elucidated, especially at organelle level.

Our previous studies have demonstrated that MSCs do have significant effects on ameliorating endothelial dysfunction and dyslipidaemia in STZ‐induced diabetic rats.^4^ Hepatic steatosis is considered as a 'benign' condition closely linked to impaired insulin action and hyperglycaemia in type 2 diabetes^23^; thus, we are interested in the MSCs’ effects and potential molecular mechanism on NAFLD. Considering that dietary fatty acids essentially affect the nutrient metabolism of human body and have played a predominant role in the development and progression of NAFLD, in this study, we established NAFLD animal model by feeding SD rats with high‐fat food for 18 weeks (Table 3). Although the bodyweight showed no significant difference between the groups, the HFD group showed macrosteatosis in liver, which suggested that this animal model may mimic the progression of NAFLD in non‐obesity humans. Meanwhile, the HFD rats that received rMSCs did not develop predominant macrovesicular steatosis characterized with massive ballooning, and showed a decreased liver TG and TC content, indicating a significant reverse of fatty liver. It is noteworthy that insulin resistance is also an important character when the development of NAFLD to NASH, and consistently, the impairment of glucose tolerance, hyperlipidaemia and hyperinsulinaemia levels were detected in these HFD rats.^24^ Our results indicated that rMSC treatment dramatically reduced the fasting insulin level in serum and the compensatory hyperplasia in islets, accompanied by decreased mRNA expression of inflammation factors in liver (Figure S2), convincing that rMSCs therapeutically ameliorated liver steatosis and IR, and also had benefit to prevent the development of NASH.

**TABLE 3:** NAFLD and NASH histological scoring system and scores of all the experimental groups

Item	Definition	Score	Scores of different experimental group			
			Chow diet	HFD (16 wk)	HFD (24 wk)	HFD + MSCs (24 wk)
Steatosis Grade	<5%	0	0	2	2	1
	5%‐33%	1				
	>33%‐65%	2				
	>66%	3				
Location	Zone 3 (centrilobular)	0	0	2	2	2
	Zone 1 (periportal)	1				
	Azonal	2				
	Panacinar	3				
Microvesicular steatosis	Absent	0	0	1	1	1
	Present	1				
Fibrosis stage	None	0	0	0	0	0
	Perisinusoidal or periportal	1				
	Perisinusoidal	2				
	and periportal	3				
	Bridging fibrosis cirrhosis	4				
Inflammation Lobular inflammation	No foci	0	0	0	1	0
	<2 foci per 200 × field	1				
	2‐4 foci per 200 × field	2				
	>4 foci per 200 × field	3				
Microgranulomas	Absent	0	0	0	1	0
	Present	1				
Liver cell injury Ballooning	None	0	0	1	2	1
	Few balloon cells	1				
	Many cells/prominent ballooning	2				
Total			0	6	9	5

Previous studies demonstrated that non‐esterified saturated FFAs, such as palmitate (C16:0), are hepatotoxic because of their ability to induce hepatocyte lipoapoptosis.^15^ In agreement with previous studies, Oil Red O staining confirmed that PA stimulation increased the lipid droplets in HepG2 cells and elevated the TG content, accompanied by changes in expressions of fatty acid degradation, synthesis and transport genes. The lipogenic transcription factor SREBP1, which regulated lipogenic genes including fatty acid synthase (FAS) and stearoyl‐CoA desaturase (SCD), was induced in the liver of HFD group and the PA‐induced HepG2 cells. Previous studies have indicated the mechanisms involved in SREBP‐1c activation by ER stress include rapid degradation of its upstream inhibitor insulin‐induced gene 1 (Insig1)^25^ and direct transcriptional activation of the SREBP‐1c gene by the IRE‐1α‐XBP1 pathway.^26^ In this study, the XBP1 (XBP1 splicing) expression in the livers of HFD rats and the PA‐stimulated HepG2 cells showed no significant difference compared with control, while the ATF4 expression was increased. These results suggested that the SREBP1c activation may be triggered by the activation of PERK‐ATF4 pathway and mediated by inhibition of Insig1. Thus, the potential mechanism of MSC administration ameliorated hepatic steatosis was possibly through down‐regulation of the SREBP1 expression by the decreased ATF4 expression, resulting in a reduced lipogenesis.

Numerous studies have demonstrated that ER stress is a major contributor to NAFLD, but its role varies depending on the cause and progression of the disease. Moreover, it is important to note that each cell type responds to ER stress and activates the UPR in a unique manner.^7^, ^8^, ^27^, ^28^ As previous studies mentioned, the UPR is regulated by the three master regulators, IRE1, PERK and ATF6. Activated IRE1α splices X‐box binding protein 1 (XBP1) mRNA and then activates further downstream genes that function in ERAD such as ER‐degradation‐enhancing‐α‐mannidose‐like protein (EDEM).^28^ And the UPR adjusts the capacity of the ER to fold and remove abnormally folded proteins according to need in a dynamic way to re‐establish homeostasis.^29^ Yang et al reported a s‐nitrosylation of IRE1α in ob/ob mice and HFD–fed mice,^30^ which suggested a progressive decline in IRE1α endoribonuclease activity with reduced XBP1 splicing. Interestingly, the XBP1 splicing expression levels were significantly up‐regulated in 7‐ and 12‐week‐old ob/ob mouse livers, before a reduction was noted in 16‐week‐old mouse livers. Additionally, Wang et al reported an increased hepatic inflammation and fibrosis in 20‐week high‐fat–fed hepatocyte‐specific IRE1α knockout mice, suggesting that IRE1α may be protective in NASH progression.^31^ In our study, the XBP1 expression showed no significant difference in HFD‐fed rats compared with the control group at the 24th week (Figure 2H). Furthermore, the XBP1s showed a decrease in PA‐treated HepG2 cells (Figure 7D), but an increase in the PA‐M group. Thus, we speculate that MSC treatment may have the capability to enhance the cellular folding and degradation capacity by XBP1s, which up‐regulates gene encoding ERAD components, and promotes the adaptive response of UPR. Certainly, further prospective studies are warranted to test these hypotheses.

Lipotoxicity will ultimately result in cell death and consequently organ dysfunction. To further explore and explain the therapeutic effect of MSCs against cell death, we detected apoptosis by TUNEL assay and Annexin V & PI staining, but there was no obvious apoptosis either in livers of HFD‐fed rats or PA‐treated HepG2 cells (data not shown), whereas our previous studies have reported a programmed cell death known as 'pyroptosis' plays an important role in NAFLD development.^32^ In this study, we found that exposure of PA led to cells obviously swelling and death, accompanied by increased expressions of pyroptosis markers (Figure 8). Pyroptosis is a novel programmed cell death, identified as caspase‐1–dependent and characterized by plasma membrane rupture and release of pro‐inflammatory intracellular contents IL‐1β and IL‐18.^20^, ^33^, ^34^ These results suggested that apoptosis may be not the main form of cell death caused by lipotoxicity. As many researches have unambiguously shown MSCs have an excellent anti‐inflammatory property, we hypothesized that the ability of MSCs to modulate pyroptosis may be an important explanation for MSC‐mediated improvement of hepatic lipotoxicity. However, the detailed mechanism still needs further investigation.

The sarcoplasmic/ER Ca^2+^ ATPase (SERCA) is an intracellular membrane transporter that utilizes the free energy provided by ATP hydrolysis for active transport of Ca^2+^ ions from the cytoplasm to the lumen of ER. The importance of Ca^2+^ as a second messenger in general and as a mediator between electrical excitation and cellular contraction in heart is well established. SERCA is critical to control the Bowditch effect in the heart, to directly contribute to the intrinsic mechanisms of heart regulation.^35^ SERCA2 deficiency occurs in human Darier‐White disease leading to glucose intolerance, decreased β‐cell ER stress.^36^ SERCA also plays a fundamental role for cellular Ca^2+^ homeostasis and signalling in hepatocytes. In ob/ob mice, insulin resistance was accompanied by the SERCA injury and ER stress in the liver,^37^ while the CDN1163, which is a novel allosteric SERCA2 modulator, could decrease blood glucose and fasting insulin levels, resulting in improved glucose tolerance in ob/ob mice even in the absence of reduction in food intake, these results indicating the important role of SERCA in metabolic disorders. Consistence with previous studies, we demonstrated that the reduction of SERCA activity increased the cytosolic Ca^2+^ load and promoted apoptosis in HFD‐fed rats and PA‐induced HepG2 cells. However, the MSC treatment significantly improved the expression and function of SERCA, and intracellular calcium homeostasis was greatly restored in the PA + MSC group. And it is noteworthy that hMSC coculture significantly alleviated the ER stress and cell injury in Thp, a specific SERCA inhibitor‐induced HepG2 cells. Additionally, the SERCA2b knockdown in HepG2 cells partially abolished the MSC‐mediated protective effect on PA‐induced hepatocyte injury. These results suggested that, upon the recovery of SERCA function by hMSC treatment, ER stress was relieved, glucose homeostasis and insulin sensitivity were improved, and the hepatic lipid accumulation was attenuated.

Recent findings demonstrate that targeting ER stress and SERCA function might be a viable therapeutic approach for the treatment of NAFLD and other metabolism disorders, for the first time, we reported here that MSCs are potentially capable of ameliorating the ER stress by enhancing the SERCA activity and improving Ca^2+^ homeostasis in NAFLD. Taken that SERCA dysfunction has been linked to the development of several disorders, the present study may disclose the novel molecular mechanisms of MSC treatment in Ca^2+^ imbalance–associated diseases, including NAFLD/NASH, T2DM and cardiac disease. Technically, these interesting observations need further convincing and investigation. An important question needs to be answered is how MSCs regulate SERCA activity in hepatocyte in the context of lipotoxicity. Previous studies demonstrated that MSCs exert their effects via exosomes, including DNA, proteins/peptides, mRNA, microRNA, lipids and organelles to recipient cells.^38^ It is reported that in vivo, EVs from MSCs with miRNA‐181‐5p overexpression alleviated liver fibrosis via activation of autophagy in CCl4‐induced liver injury. In vitro, transfer of miNRA‐181‐5p from MSCs to mouse hepatic stellate cells by EV uptake was shown to be responsible for MSC‐mediated protection.^39^ Additionally, the hepatocyte growth factor (HGF) derived from conditioned culture media of MSCs has been demonstrated playing an important role in calcium homeostasis and ER stress in fibrotic lungs.^40^ We assume that the paracrine activity of MSCs is essential for their therapeutic functions, and the exact mechanisms are warranted to be investigated in more details.

In conclusion, we established NAFLD rats and PA‐induced hepatocytes to investigate the effects of MSC therapy on steatosis, insulin resistance and dyslipidaemia, and explored the potential molecular mechanisms. Our findings demonstrated that MSC treatment restored the intracellular Ca^2+^ homeostasis by regulating SERCA, contributing to the alleviation of ER stress and improvement of metabolic dysfunction in NAFLD rats and PA‐induced HepG2 cells. We reaffirmed that ER stress plays a critical role in the pathology of NAFLD, and SERCA could be an interesting target for metabolic dysfunction. Our study suggested that MSC treatment may be considered as a novel and promising therapeutics in future to benefit the Ca^2+^ homeostasis and ER stress–related disorders.

## CONFLICT OF INTEREST

We declare that we have no financial and personal relationships with other people or organizations that can inappropriately influence our work; there is no professional or other personal interest of any nature or kind in any product, service and/or company that could be construed as influencing the position presented in, or the review of, this paper.

## AUTHOR CONTRIBUTION

**Linzhao Li:** Conceptualization (equal); Data curation (lead); Formal analysis (lead); Funding acquisition (equal); Investigation (lead); Methodology (equal); Project administration (equal). **Xin Zeng:** Conceptualization (equal); Data curation (equal); Formal analysis (equal); Funding acquisition (equal); Investigation (equal); Methodology (equal). **Zhenzhen Liu:** Investigation (equal); Methodology (equal). **Xuanming Chen:** Formal analysis (equal); Investigation (equal); Methodology (equal). **Lan Li:** Investigation (supporting); Methodology (supporting). **Ruixi Luo:** Investigation (supporting); Methodology (supporting). **Xiaohong Liu:** Investigation (supporting); Methodology (supporting). **Jie Zhang:** Methodology (supporting). **Jingping Liu:** Conceptualization (equal); Project administration (equal). **Yanrong Lu:** Conceptualization (equal); Project administration (equal). **Jingqiu Cheng:** Conceptualization (equal); Project administration (equal). **Younan Chen:** Conceptualization (lead); Data curation (equal); Formal analysis (equal); Funding acquisition (equal); Investigation (equal); Methodology (equal); Project administration (lead).

## Supporting information

Figure S1

Figure S2

Supplementary Material

Method S1
