# Supplementary figures and images for: Mesenchymal stromal cells protect hepatocytes from lipotoxicity through alleviation of endoplasmic reticulum stress by restoring SERCA activity
Source: J Cell Mol Med. 2021 Feb 16;25(6):2976–93. doi: 10.1111/jcmm.16338 (PMC7957164; doi:10.1111/jcmm.16338)

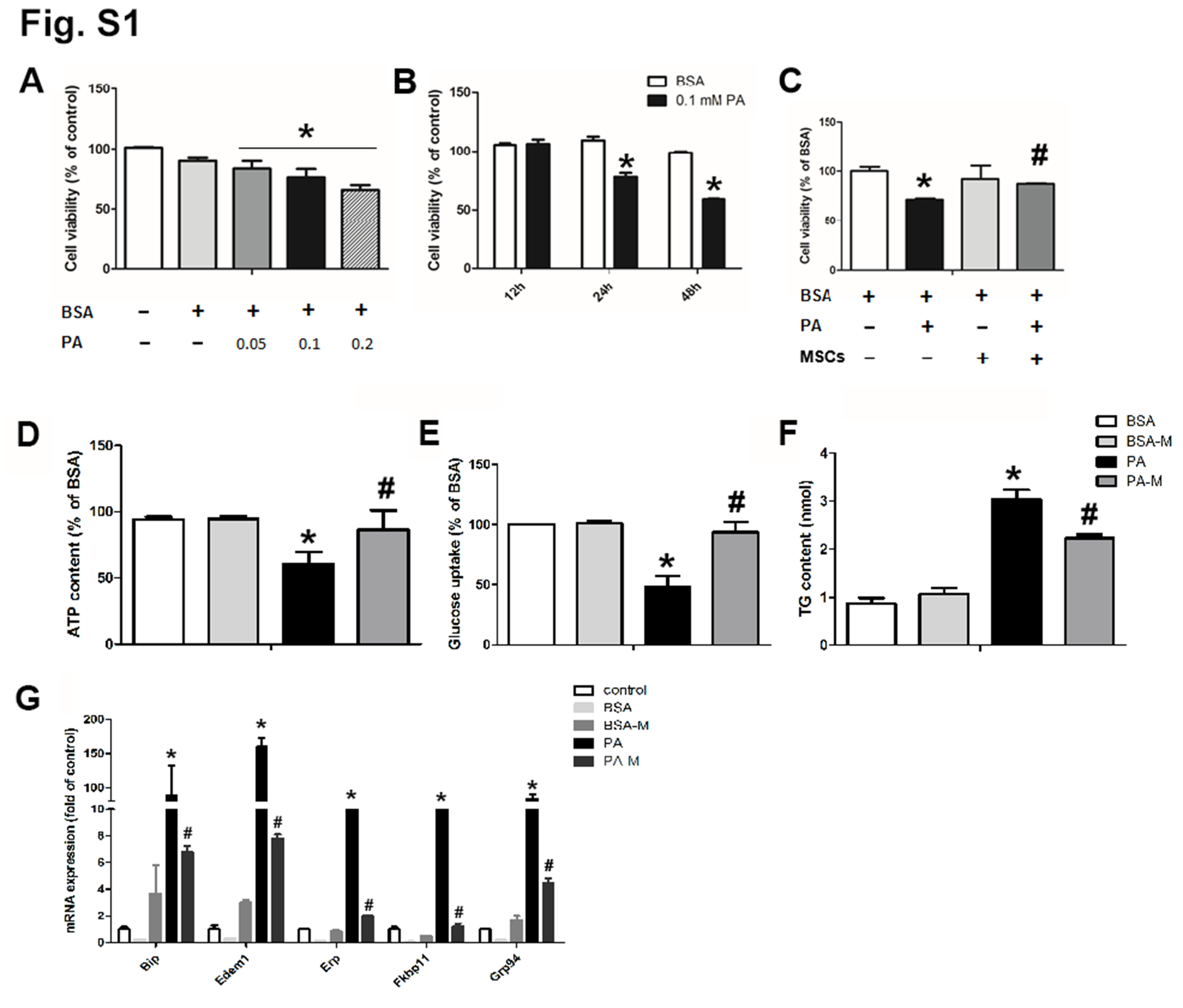

Supplement: Supplementary file 1 — Figure S1 [file JCMM-25-2976-s003.tif]

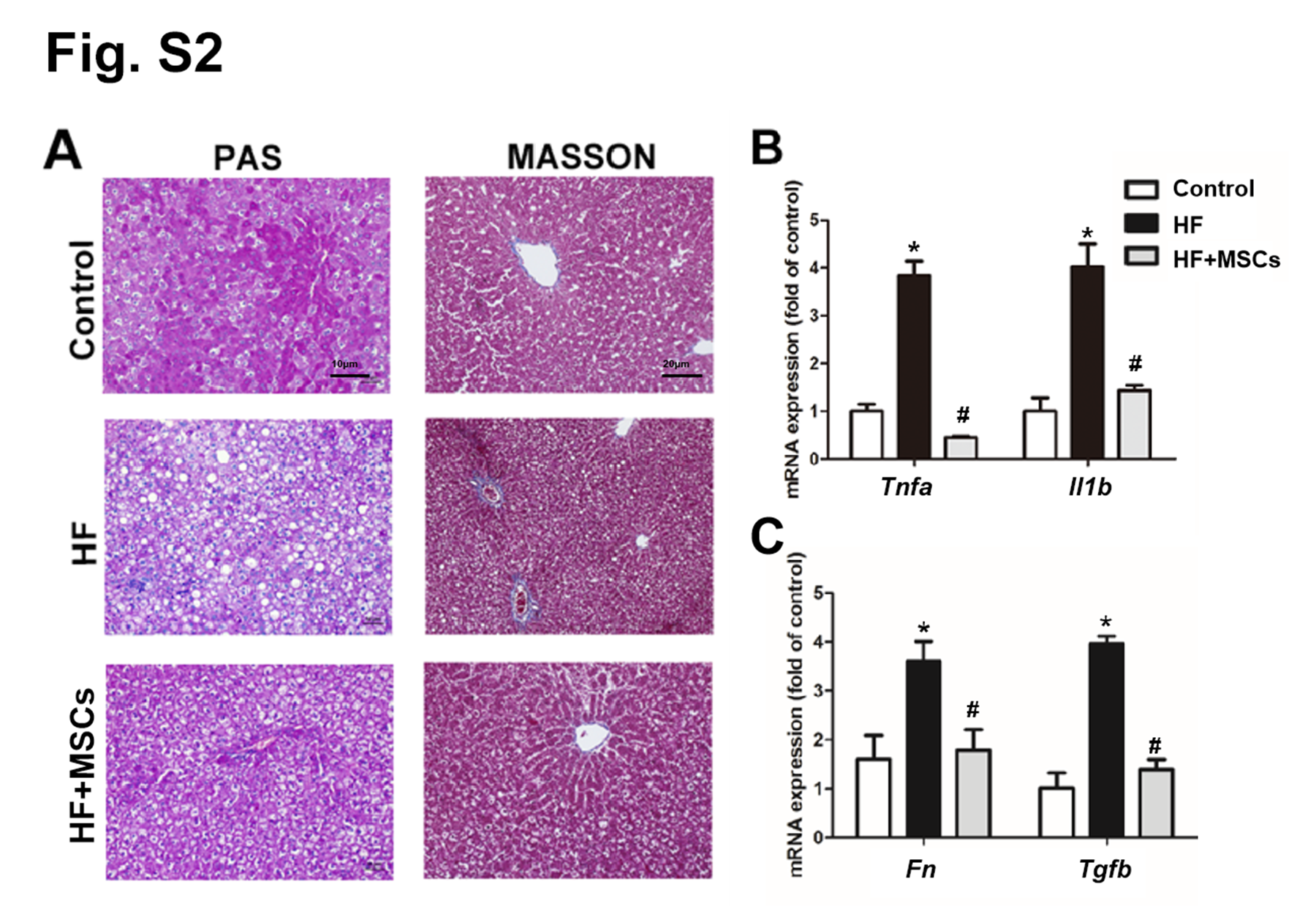

Supplement: Supplementary file 2 — Figure S2 [file JCMM-25-2976-s001.tif]
