# Supplementary material for: Mesenchymal stromal cells protect hepatocytes from lipotoxicity through alleviation of endoplasmic reticulum stress by restoring SERCA activity
Source: J Cell Mol Med. 2021 Feb 16;25(6):2976–93. doi: 10.1111/jcmm.16338 (PMC7957164; doi:10.1111/jcmm.16338)
Supplement: Supplementary file 3 — Supplementary Material [file JCMM-25-2976-s004.docx]

Supplemental Figures

Figure S1

Fig. S1 Rat bone marrow-derived MSCs ameliorated PA-induced injuries in primary rat-hepatocytes. Time and dose dependent toxicity of PA on primary rat-hepatocytes (A, B). Primary rat-hepatocyte were incubated with 0.05 mM BSA or PA and co-cultured with or without rMSCs, and the cell viability (C), cellular total ATP content (D), cellular glucose utilization (E) and TG content (F) were measured after 24 hours. The mRNA expression levels of ER stress markers (G) were detected by q-PCR after 24 hours treatment. Results are presented as means ± SEM from three independent experiments, *P<0.05vs. the control or BSA group; ^#^P<0.05 vs. the PA group.

Figure S2

Fig. S2 Systemic MSCs transplantation altered the gene expressions of inflammation and fibrosis markers molecular PAS and MASSON staining of liver sections in the three groups (A). The mRNA levels of inflammation markers *Tnfa* and *Il1b*(B), and fibrosis markers *Fn* and *Tgfb* in the liver (B and C). The data are expressed as mean ± SEM, *P<0.05 vs. the control group; ^#^P<0.05 vs. the HFD group.
