# Supplementary material for: Mesenchymal stromal cells protect hepatocytes from lipotoxicity through alleviation of endoplasmic reticulum stress by restoring SERCA activity
Source: J Cell Mol Med. 2021 Feb 16;25(6):2976–93. doi: 10.1111/jcmm.16338 (PMC7957164; doi:10.1111/jcmm.16338)
Supplement: Supplementary file 4 — Method S1 [file JCMM-25-2976-s002.docx]

**Supplemental Materials and Methods:**

Primary rat hepatocytes isolation and culture

Neonatal rat primary hepatocytes were isolated from 3 days old Sprague–Dawley (SD) rats, the liver tissues of 5 SD neonatal rats were cut into 1mm^3^ size in precooling DMEM. After discarding the blood clots, tissues were transferred to 0.125% trypsin solution for overnight digestion at 4°C. Cell suspension was centrifuged at 240, 30,10×g for 5 min at 4°C, then cell pellet was re-suspended in 20 ml warm complete medium. After seeding cells in a T75 ﬂask for 24h, the medium was changed at 2 days intervals.
